# A segregated reduced-order model of a pressure-based solver for turbulent compressible flows

**DOI:** 10.1186/s40323-025-00284-8

**Published:** 2025-02-19

**Authors:** Matteo Zancanaro, Valentin Nkana Ngan, Giovanni Stabile, Gianluigi Rozza

**Affiliations:** 1 Mathematics Area, mathLab, SISSA, via Bonomea 265, I-34136 Trieste, Italy; 2 The Biorobotics Institute, Sant’Anna School of Advanced Studies, Viale Rinaldo Piaggio 34, 56025 Pontedera, Pisa Italy

**Keywords:** Compressible flows, Reduced-order model, SIMPLE algorithm, Turbulence, Neural networks

## Abstract

This article provides a reduced-order modelling framework for turbulent compressible flows discretized by the use of finite volume approaches. The basic idea behind this work is the construction of a reduced-order model capable of providing closely accurate solutions with respect to the high fidelity flow fields. Full-order solutions are often obtained through the use of segregated solvers (*solution variables are solved one after another*), employing slightly modified conservation laws so that they can be decoupled and then solved one at a time. Classical reduction architectures, on the contrary, rely on the Galerkin projection of a complete Navier–Stokes system to be projected all at once, causing a mild discrepancy with the high order solutions. This article relies on segregated reduced-order algorithms for the resolution of turbulent and compressible flows in the context of physical and geometrical parameters. At the full-order level turbulence is modeled using an eddy viscosity approach. Since there is a variety of different turbulence models for the approximation of this supplementary viscosity, one of the aims of this work is to provide a reduced-order model which is independent on this selection. This goal is reached by the application of hybrid methods where Navier–Stokes equations are projected in a standard way while the viscosity field is approximated by the use of data-driven interpolation methods or by the evaluation of a properly trained neural network. By exploiting the aforementioned expedients it is possible to predict accurate solutions with respect to the full-order problems characterized by high Reynolds numbers and elevated Mach numbers.

## Introduction

In the last decades, fluid flow simulations have progressively enlarged their applicability and their influence in many different research fields (general overviews can be found in [1–3]). Nowadays, applications of computational fluid dynamics (CFD) have reached widespread application areas such as, shape optimization for naval/automotive/aerospace engineering [4, 5], cardiovascular in real time surgery [6], chemistry industrial processes [7, 8] or weather forecasts [9]. As the demand for usability and reliability in CFD methods increases, current hardware designs are becoming insufficient to meet the computational requirements in a timely manner. As a result, a considerable amount of CFD research is focused on developing new, efficient techniques to reduce computation times. This challenge frequently arises in applications such as shape optimization problems, uncertainty quantification studies, and optimal control frameworks.

To address this issue, various strategies have been explored recently. One of such approach is Galerkin projection, which has been widely applied to develop new reduction techniques that offer efficient, accurate, and more cost-effective solutions for varying parameter selections. This method leverages information from only a few full-order solutions across different parameter values. In fact, in a reduced-order model (ROM) framework, parameters and parametric studies are central to understanding the influence of different variables on the system’s behavior while maintaining the computational efficiency offered by ROMs. A reduced-order model is a simplified representation of a complex, high-dimensional system, designed to capture the essential behavior and dynamics of the system with significantly reduced computational effort. By focusing on the most important modes or features of the full-order model, ROMs provide fast and efficient simulations while maintaining accuracy within an acceptable range for various analyses. The most commonly used technique in ROMs is Galerkin projection. In Galerkin projection, the original high-dimensional problem is projected onto a lower-dimensional subspace. This significantly reduces computational complexity while preserving the system’s dominant behavior [10]. For fluid flow applications, relevant studies can be found in [11–15]. There are numerous options for leveraging the dynamic content included in high-fidelity systems. The most commonly used one is the Proper Orthogonal Decomposition (POD) [16–21], the Proper Generalized Decomposition (PGD) [22, 23], the Dynamic Mode Decomposition (DMD) [24, 25]. The initial concept of the POD, which was first developed in the domain of fluid dynamics to examine turbulence, is to decompose a vector field into a series of deterministic spatial functions weighted by time/parameter coefficients.

In recent years, while the concept of machine learning (ML) is not new, its applications within the fluid dynamics community have significantly expanded. This growth is driven by advancements in algorithms, increased computational power, more affordable memory, and the availability of vast amounts of data. As a result, ML has emerged as a prominent area of research in the field. Leveraging ML algorithms has made solving complex, non-linear parametric partial differential equations (PDEs) more efficient and accessible than ever before. Numerous approaches for applying ML to CFD problems have been investigated in the literature, as demonstrated in several studies, for instance in [26–37] to name few. For example, the combination of the POD and neural networks (NNs) has been applied across a wide range of cases, including the non-linear Poisson equation in one and two spatial dimensions, as well as two-dimensional cavity flows governed by the steady incompressible Navier–Stokes equations. Both POD-based projection techniques and ML methods, offer valuable insights but also have their limitations. Projection techniques are closely aligned with the physical laws of the problem, using modal basis functions derived from real solutions to capture the primary dynamics. These modes are then used to project and reconstruct the solutions of conservation equations on reduced manifolds. However, challenges arise in dealing with non-linearity and the non-affine nature of parameterized formulations, which can complicate their application. Additionally, projection methods may not be feasible if the governing equations are inaccessible, such as in commercial software where the underlying laws are not fully disclosed. In contrast, ML techniques offer greater versatility. They require only a set of pre-trained solutions and are independent of the complexity of the problemâ€™s mathematical formulation. These methods are designed to produce accurate approximations rapidly. However, a significant drawback is their weaker connection to the underlying physics, making it difficult to interpret the individual components of the ML architecture in terms of physical phenomena. For this reason, they may give inaccurate results thanks to impossibility in having a deeper check on networks responses.

Taking all the aforementioned examinations under consideration, this work applies the new mixed technique [26] on compressible Navier–Stokes problems, which is capable of merging the advantages of projection techniques together with data-driven architectures. In particular, in our approach, classical projection methods are used for the Favre Averaged Navier Stokes (FANS) equations while a neural network gets trained to provide the eddy viscosity solutions in a turbulence modelling approach. These new contributions result to a reduced-order models that are independent of the selection of turbulence models for any segregated solvers for compressible flows capable to reduce the computational cost associated with fluid flow problems characterized by high Reynolds numbers and elevated Mach numbers. The main goal is to propose an architecture proficient in dealing with different types of parametrizations for compressible flows. Moreover, one of the most relevant focuses concerning this work is constituted by a coherent approach between full-order and reduced-order solutions, by developing a new reduced compressible SIMPLE (Semi-Implicit Method for Pressure Linked Equations) algorithm.

This manuscript is structured as follows: The section “The compressible Navier–Stokes equations” presents the equations used in this work; subsection “Proper Orthogonal Decomposition procedure” explains the POD procedure employed to obtain the modal basis functions. In subsection “Reduced-SIMPLE algorithm for compressible flows” the core algorithm used for our technique is introduced together with subsection “Turbulence treatment” where the AI architecture for turbulence treatment is shown. Two different test cases, a physically parameterized and a geometrically parameterized ones, are exposed in subsection “Physical parametrization test case” and subsection “Geometrical parametrization test case” respectively. Finally, in subsection “Conclusions and future perspectives”, few considerations on the results and some possible developments for this work are presented.

## The compressible Navier–Stokes equations

In this work, we want to deal with parameterized compressible Navier-Stokes equations problems. To manage the compressibility of the fluid, we selected a common strategy for this kind of applications: the Favre averaging. The equations describing the physics are the following ones:

1 $$\begin{aligned}&\displaystyle \frac{\partial \rho }{\partial t} + \nabla \cdot \left( \rho \varvec{u}\right) = 0 ~~ \text {in} \ \Omega (\pi ), \end{aligned}$$

2 $$\begin{aligned}&\quad \displaystyle \frac{\partial \rho \varvec{u} }{\partial t} + \nabla \cdot \left[ \rho \varvec{u} \otimes \varvec{u}+ p \varvec{I} - \varvec{\tau } \right] = 0 ~~ \text {in} \ \Omega (\pi ), \end{aligned}$$

3 $$\begin{aligned}&\quad \displaystyle \frac{\partial \rho e_0}{\partial t} + \nabla \cdot \left[ \rho \varvec{u}e_0 + p\varvec{u} - \varvec{u} \cdot \varvec{\tau } + \varvec{q}\right] = 0 ~~ \text {in} \ \Omega (\pi ), \end{aligned}$$

4 $$\begin{aligned}&\quad \varvec{u} = \varvec{g}_D ~~ \text {in} ~ \Gamma _D, \end{aligned}$$

5 $$\begin{aligned}&\quad \nu \displaystyle \frac{\partial \varvec{u}}{\partial \varvec{n}}-p \varvec{n} = \varvec{g}_N ~~\text {in} ~ \Gamma _{N}, \end{aligned}$$

where $\rho$ represent the density, $\varvec{u}$ the flow velocity, *p* the pressure, $\varvec{\tau }$ the viscous stress tensor, $e_0$ the total energy, and $\varvec{I}$ the identity tensor. The boundary conditions include $\Gamma _D$, where Dirichlet conditions $\varvec{g}_D$ are applied, and $\Gamma _N$, where Neumann conditions $\varvec{g}_N$ are imposed. Here, $\nu$ refers to the kinematic viscosity, and $\varvec{n}$ is the unit normal vector. The computational domain is denoted by $\Omega (\pi )$, which, in cases of geometric parametrization, can depend explicitly on the parameter $\pi$. The heat-flux $\varvec{q}$ is given by Fourier’s law:

6 $$\begin{aligned} \varvec{q} =\lambda \nabla T \equiv C_p\frac{\mu }{Pr}\nabla T, \end{aligned}$$

with the laminar Prandtl number *Pr* is given by $Pr = \frac{C_p\mu }{\lambda }$. To close these equations, it is also necessary to specify an equation of state. Assuming air to be an ideal gas, the following relations are valid:

$$\begin{aligned} \gamma \equiv C_p/C_v, ~~ p = \rho R T, ~~ e = C_vT, ~~ C_p - C_v = R. \end{aligned}$$

Being *R* the gas constant, $C_v$ is the constant volume, and $C_p$ means specific heat at constant pressure, $\gamma$ is the adiabatic index, *e* the internal energy, and *T* the temperature. In the Favre Averaged Navier–Stokes (FANS) equations, all the variables (density $\rho$, pressure *p*, velocity $\varvec{u}$, total energy $e_0$, temperature *T* and internal energy *e*) are decomposed in an averaged part and a fluctuating one as follows:

7 $$\begin{aligned}&\rho = \overline{\rho } + \rho ', ~~~ p = \overline{p} + p', ~~~ T = \tilde{T} + T'' \end{aligned}$$

8 $$\begin{aligned}&\quad e_0 = \tilde{e_0} + e_0'', ~~~ \varvec{u} = \tilde{\varvec{u}} + \varvec{u}'', ~~~ e = \tilde{e} + e'' . \end{aligned}$$

Superscript $\tilde{\square }$ indicates the Favre averaging which correspond to a density weighted Reynolds averaging $\overline{\square }$. Given a certain variable $\Phi (t)$, we have:

9 $$\begin{aligned} \overline{\Phi }&= \frac{1}{T} \int _T \Phi (t) dt \Rightarrow \Phi ' = \Phi - \overline{\Phi } \end{aligned}$$

10 $$\begin{aligned} \tilde{\Phi }&= \frac{\overline{\rho \Phi }}{\overline{\rho }} \Rightarrow \Phi '' = \Phi - \tilde{\Phi } . \end{aligned}$$

Plugging Eq. 7, Eq. 8, Eq. 9 and Eq. 10 in Eq. 1, Eq. 2, Eq. 3 lead to:

11 $$\begin{aligned}  &   \displaystyle \frac{\partial \overline{\rho }}{\partial t} + \nabla \cdot \left( \overline{\rho } \tilde{\varvec{u}}\right) = 0 ~ \text {in} ~ \Omega (\pi ), \end{aligned}$$

12 $$\begin{aligned}  &   \quad \displaystyle \frac{\partial \overline{\rho } \tilde{\varvec{u}}}{\partial t} + \nabla \cdot \left[ \overline{\rho } \tilde{\varvec{u}} \otimes \tilde{\varvec{u}} - \tilde{\varvec{\tau }}_{turb} - \tilde{\varvec{\tau }} + \overline{p} \varvec{I}\right] = 0 ~~ \text {in} ~ \Omega (\pi ), \end{aligned}$$

13 $$\begin{aligned}  &   \quad \displaystyle \frac{\partial \overline{\rho } \tilde{e}_0}{\partial t} + \nabla \cdot \left[ \overline{\rho } \tilde{\varvec{u}} \tilde{e}_0 - C_p \frac{\alpha _{eff}}{\gamma }\nabla \tilde{T} \right] + \nabla \cdot \left[ \overline{p} \tilde{\varvec{u}} - \tilde{\varvec{u}} \cdot \tilde{\varvec{\tau }} - \tilde{\varvec{u}} \cdot \tilde{\varvec{\tau }}_{turb} \right] = 0 ~~ \text {in} ~ \Omega (\pi ), \end{aligned}$$

14 $$\begin{aligned}  &   \quad \tilde{\varvec{u}} = \varvec{g}_D ~ \text {in}~ \Gamma _D, \end{aligned}$$

15 $$\begin{aligned}  &   \quad \nu \displaystyle \frac{\partial \tilde{\varvec{u}}}{\partial \varvec{n}} - \overline{p} \varvec{n} = \varvec{g}_N ~\text {in} ~\Gamma _{N}, \end{aligned}$$

with $\alpha _{eff} = \gamma \bigg ( \frac{\mu }{Pr} + \frac{\mu _t}{Pr}_{t} \bigg )$, and where $\overline{p}$, $\tilde{\varvec{u}}$ and $\tilde{e}$ become the unknowns of the problem. $\mu$ is the dynamic viscosity, $\mu _t$ is the eddy viscosity owing to turbulence, *Pr* indicates the Prandtl number and $Pr_t$ its turbulent counterpart which is a constant value. The molecular $\tilde{\varvec{\tau }}$ and Reynolds-Stress $\tilde{\varvec{\tau }}_{turb}$ tensors are given by:

16 $$\begin{aligned} \tilde{\varvec{\tau }} = 2\mu \tilde{\varvec{S}}, \hspace{0.05cm} \tilde{\varvec{\tau }}_{turb} = 2\mu _t \tilde{\varvec{S}} -\frac{2}{3}\bar{\rho }k\varvec{I}, \end{aligned}$$

where $\tilde{\varvec{S}} = \frac{\nabla \tilde{\varvec{u}} + \nabla \tilde{\varvec{u}}^\intercal }{2} - \frac{1}{3} \nabla \cdot \tilde{\varvec{u}} \varvec{I}$, and $k = \widetilde{\frac{\varvec{u}'' \cdot \varvec{u}''}{2}}$. Moreover, the density averaged total energy $\tilde{e}_0$ is rewritten in the internal energy form:

17 $$\begin{aligned} \tilde{e}_0 = \tilde{e} + \frac{\tilde{\varvec{u}} \cdot \tilde{\varvec{u}}}{2} + k, \end{aligned}$$

Eq. 12, and Eq. 13 are obtained after some approximations and assumptions from an eddy viscosity point of view. The reader interested in the averaging procedure and modelling should refer to [38].

From now on, Eq. 11, Eq. 12, and Eq. 13 will be considered only in its steady-state formulation. All the averaged variables are dependent on the parameter $\pi$ but, for the sake of simplicity, the following notation will be used:

$$\begin{aligned} \overline{\rho } = \overline{\rho }(\pi ), \hspace{0.1cm} \overline{p} = \overline{p}(\pi ), \hspace{0.1cm} \tilde{\varvec{u}} = \tilde{\varvec{u}}(\pi ), \hspace{0.1cm} \tilde{T} = \tilde{T}(\pi ), \hspace{0.1cm} \tilde{e} = \tilde{e}(\pi ). \end{aligned}$$

In the energy equation, the viscous terms are neglected in many solvers. This, can be reasonably true if compared with the other terms present into the energy equation. Moreover, the turbulent kinetic energy is neglected in the total energy. This results in the following system:

18 $$\begin{aligned}&\nabla \cdot \left( \overline{\rho } \tilde{\varvec{u}}\right) = 0 ~~~ \text {in} \ \Omega (\pi ), \end{aligned}$$

19 $$\begin{aligned}&\quad \displaystyle \nabla \cdot \left[ \overline{\rho } \tilde{\varvec{u}} \otimes \tilde{\varvec{u}} - \mu _{eff} \bigg ( \nabla \tilde{\varvec{u}} + \nabla \tilde{\varvec{u}}^\intercal - \frac{2}{3} \nabla \cdot \tilde{\varvec{u}} \varvec{I}\bigg ) + \overline{p} \varvec{I}\right] = 0 ~~~ \text {in} \ \Omega (\pi ), \end{aligned}$$

20 $$\begin{aligned}&\quad \displaystyle \nabla \cdot \bigg [ \overline{\rho } \tilde{\varvec{u}} \bigg ( \tilde{e} + \frac{\tilde{\varvec{u}} \cdot \tilde{\varvec{u}}}{2}\bigg ) - \alpha _{eff} \nabla \tilde{e} + \overline{p} \tilde{\varvec{u}} \bigg ] = 0 ~~~ \text {in} \ \Omega (\pi ), \end{aligned}$$

21 $$\begin{aligned}&\quad \tilde{\varvec{u}} = \varvec{g}_D ~~~ \text {in} ~ \Gamma _D, \end{aligned}$$

22 $$\begin{aligned}&\quad \nu \displaystyle \frac{\partial \tilde{\varvec{u}}}{\partial \varvec{n}} - \overline{p} \varvec{n} = \varvec{g}_N ~~~ \text {in} ~ \Gamma _{N}. \end{aligned}$$

With $\mu _{eff} = \mu + \mu _t$, and $\alpha _{eff} = \gamma \bigg ( \frac{\mu }{Pr} + \frac{\mu _t}{Pr}_{t} \bigg )$. It is now clear that all the turbulence-related terms of the equations rely on $\mu _t$ to be calculated. For this reason, since only the eddy viscosity is required, a common 2-equations turbulent model as, e.g., $k-\epsilon$ or $k-\omega$ [38], is sufficient as a closure for the problem.

## Reduced-order modelling architecture

This part of the manuscript presents the POD in a nutshell, after that, addresses the reduced-SIMPLE algorithm and finally the treatment of the eddy viscosity when dealing with turbulence flows.

### Proper orthogonal decomposition procedure

The scope of this work is to find an efficient and reliable reduced-order model to be able to solve the full-order model for many different values of the parameter $\pi$ without solving the Finite Volume discretized equations every time from scratch. For this reason, we developed a new procedure based on a POD-Galerkin scheme.

The whole machinery is divided in two main steps: an offline phase which consists on the resolution of a certain number $N_{\pi }$ of full-order solutions, trying to extract as much information as possible from this set, and an online phase consisting on the resolution of a dimensionally reduced problem for all the different needed parametric configurations. What is new in this method is to be capable of resulting as general as possible with respect to the selected full-order turbulence model and, at the same time, as coherent as possible with respect to high fidelity solutions.

Let $\mathbb {P} = \{ \pi _1, \ldots , \pi _{N_{\pi }} \}$ be the training parameters set. For every parameter $\pi _i \in \mathbb {P}$, the full-order problem can be solved to obtain the corresponding solution $\varvec{s}_i$. All these offline solutions are then stored in the snapshots matrix:

$$\begin{aligned} \varvec{S} = \begin{bmatrix} s_{11} &  s_{21} &  \dotsc &  s_{{N{\pi }}_1}\\ \vdots &  \vdots &  \vdots &  \vdots \\ s_{1{N_h}} &  s_{2{N_h}} &  \dotsc &  s_{{N_{\pi }}{N_h}}\\ \end{bmatrix}. \end{aligned}$$

**Fig. 1:**
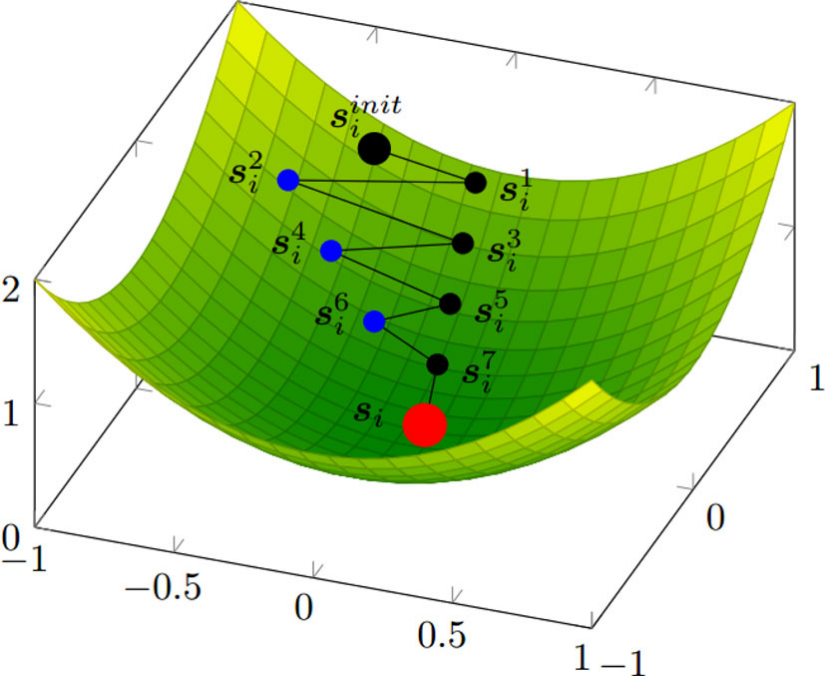
Scheme of the snapshots selection for $\Delta = 2$: black dots are discarded intermediate solutions, blue dots are saved intermediate solutions while the red dot represents the final solution

In our case we want to construct an online solver able to mimic the offline convergence dynamics. For this reason the use of a monolithic (*non-segregated*) approach for the reduced problem is not a good choice as the *offline* solutions are obtained relying on a segregated solver; also at the *online* level a segregated strategy has to be applied to obtain solutions which are as consistent as possible. For a discussion on a similar consistent approach in the context of explicit time integration schemes, the reader is referred to [39]. To obtain an algorithm able to properly follow the behaviour of the high fidelity algorithm, the set of snapshots is enriched by adding a certain amount of intermediate solutions $\varvec{s}_i^j$ obtained during the offline iterations. The distance between exported intermediate solutions is set to $\Delta$ as shown in Fig. 1. Since the solution fields during these iterations vary a lot, from the first attempt for the variables to the last resolution, the information contained into the converged snapshots is not sufficient to ensure the correct reduced reconstruction of the path to the global minimum for Eq. 1, Eq. 2, Eq. 3. By adding some non-physical solutions to the snapshots’ matrix, which is what is happening by inserting non-converged fields, we are somehow polluting the physical content, but the convergence properties of the algorithm are *quite acceptable* in any case. To reach a balance between convergence and reliability, $\Delta$ can be varied and the total amount $N_{int}$ of selected intermediate solutions can be modified. The new snapshots matrix then reads:

$$\begin{aligned} \varvec{S} = \left[ \varvec{s}_1^1, \varvec{s}_1^2, \ldots , \varvec{s}_1^{N_{int}}, \varvec{s}_1, \dots , \varvec{s}_{N_{\pi }}^1, \varvec{s}_{N_{\pi }}^2, \cdots , \varvec{s}_{N_{\pi }}^{N_{int}}, \varvec{s}_{N_{\pi }}\right] , \end{aligned}$$

where $\varvec{s}_i^j$ is the solution obtained at the $(j \, \Delta )$-th iteration for the *i*-th offline parameter.

In a POD-Galerkin approach, the reduced order solution $\varvec{s}^r$ is obtained as a linear combination of some pre-calculated basis functions $\varvec{\xi }$:

23 $$\begin{aligned} \varvec{s}^r(\varvec{x}, \pi ) = \sum _{i=1}^{N_r} \beta _i(\pi ) \varvec{\xi }_i(\varvec{x}), \end{aligned}$$

where $N_r < N_{\pi }$ is the number of basis functions to be used for the reconstruction and the $\beta _i$ are the coefficients depending only on the parameter representing the reduced solution.

Once provided a certain amount $N_t$ of high fidelity solutions, with $N_t > N_{\pi }$ because of the intermediate snapshots, the best reduced order model we can get is the one able to fully reproduce the training offline solutions with no error with respect to it. Of course this is not achievable but we would like the $L^2$ norm of the error $E_{ROM}$ between all the offline solutions and the respective online ones to be as low as possible:

$$\begin{aligned} E_{ROM} = \sum _{i=1}^{N_t} ||\varvec{s}^{ROM}_i - \varvec{s}_i ||_{L^2} = \sum _{i=1}^{N_t} \Bigg |\Bigg | \sum _{j=1}^{N_r} \beta _j(\pi ) \varvec{\xi }_j(\varvec{x}) - \varvec{s}_i \Bigg | \Bigg |_{L^2}. \end{aligned}$$

It is well known (see, e.g., [19]) that the basis functions best performing in this sense are the ones obtained through a Proper Orthogonal Decomposition (POD) applied to the snapshots matrix $\varvec{S}$. The eigen problem

$$\begin{aligned} \varvec{C} \varvec{V} = \varvec{V} \varvec{\lambda }, \end{aligned}$$

has to be resolved, where $\varvec{C} \in \mathbb {R}^{N_t \times N_t}$ is the correlation matrix containing all the inner products in the form $(\varvec{s}_i, \varvec{s}_j)_{L^2(\Omega )}$. $\varvec{V} \in \mathbb {R}^{N_t \times N_t}$ is the matrix containing its eigenvectors while $\varvec{\lambda }$ is the diagonal matrix containing the eigenvalues.

The basis functions are then constructed as just a linear combination of the snapshots contained in $\varvec{S}$:

$$\begin{aligned} \varvec{\xi }_i(\varvec{x}) = \frac{1}{{N_t\sqrt{\lambda _i}}} \sum _{j=1}^{N_t} \varvec{V}_{ji} \varvec{s}_j (\varvec{x}). \end{aligned}$$

The basis functions matrix is then defined as:

$$\begin{aligned} \varvec{\Xi } = \left[ \varvec{\xi }_1, \cdots , \varvec{\xi }_{N_r} \right] \in \mathcal {R}^{N_h \times N_r}. \end{aligned}$$

The interested reader may refer to [21, 40, 41] for a detailed explanation regarding POD approaches.

### Reduced-SIMPLE algorithm for compressible flows

In this paragraph, we will discuss the reduced algorithm developed for the resolution of compressible flows where no discontinuities are present. This means it is only suited for subsonic cases where the Mach number is lower than 1 in all the points of the domain. In this paragraph, the reduced algorithm is based on the SIMPLE algorithm. In the following, it is assumed that all the equations: continuity, momentum and energy equations are discretized and written in the following form:

24 $$\begin{aligned}&\textbf{A}_u \varvec{u}_h = \varvec{b}_u,\quad \textbf{B}_p \varvec{p}_h = \varvec{b}_p, \quad \textbf{E}_e \varvec{e}_h = \varvec{b}_e ~ \text {where}; \end{aligned}$$

25 $$\begin{aligned}&\quad \textbf{A}_u \in \mathbb {R}^{dN_h\times dN_h}, \quad \textbf{B}_p \in \mathbb {R}^{N_h\times N_h}, \quad \text {and} \quad \textbf{E}_e \in \mathbb {R}^{N_h\times N_h}, \end{aligned}$$

indicate the matrices containing the terms related to velocity, pressure, and energy for the discretized continuity, momentum, and energy equations respectively.

26 $$\begin{aligned} \textbf{b}_u \in \mathbb {R}^{dN_h}, \quad \textbf{b}_p\in \mathbb {R}^{N_h}, \quad \text {and} \quad \textbf{b}_e\in \mathbb {R}^{N_h}; \end{aligned}$$

are the related source terms. In addition, $\varvec{u}_h$, $\varvec{p}_h$, and $\varvec{e}_h$ are the vectors where all the $\tilde{\varvec{u}}_i$, ${\bar{p}}_i$, and ${\tilde{e}}_i$ variables are collected with $d=2$ the dimension of the computational domain and $N_h$ being the number of control volumes (cells) in the mesh. In the following, Galerkin projection (on the fully discrete equations) is used for the construction of the reduced-order method. We assume the following decompositions introduced in section “Proper Orthogonal Decomposition procedure”

27 $$\begin{aligned} \varvec{u}_h = \sum _{i=1}^{N_u} a_i (\cdot ) \varvec{\phi }_i (\varvec{x}) = \varvec{\Phi } \varvec{a}^\intercal ,~ \varvec{p}_h = \sum _{i=1}^{N_p} b_i (\cdot ) \varvec{\xi }_i (\varvec{x}) = \varvec{\Xi } \varvec{b}^\intercal ,~ \varvec{e}_h = \sum _{i=1}^{N_e} c_i (\cdot ) \varvec{\theta }_i (\varvec{x}) = \varvec{\Theta } \varvec{c}^\intercal . \end{aligned}$$

Where $\tilde{\varvec{u}}_r \simeq \varvec{u}_h$, $\overline{p}_r \simeq \varvec{p}_h$, and $\tilde{e}_r \simeq \varvec{e}_h$. Similarly, $a_i(\cdot )$, $b_i(\cdot )$, and $c_i(\cdot )$ are modal coefficients which can time, parameters dependent or both. $\varvec{\phi }_i$, $\varvec{\xi }_i$, and $\varvec{\theta }_i$ are the basis functions corresponding to the POD modes of the velocity, pressure, and energy fields stored respectively in $\varvec{\Phi } \in \mathbb {R}^{dN_h\times N_u}$, $\varvec{\Xi } \in \mathbb {R}^{N_h\times N_p}$, and $\varvec{\Theta } \in \mathbb {R}^{N_h\times N_e}$. In addition, $N_u$, $N_p$, and $N_e$ being the numbers of basis functions selected for the predicted velocity, pressure, and energy solutions respectively. $\varvec{a} \in \mathbb {R}^{N_u}$, $\varvec{b} \in \mathbb {R}^{N_p}$, and $\varvec{c} \in \mathbb {R}^{N_e}$ are the vectors containing the coefficients for the velocity expansion while the same reads for pressure and energy.

The linear systems in eq. (24) are projected using the respective basis functions defined in eq. (27) respectively leading to:

28 $$\begin{aligned} \varvec{A}_u^r\varvec{a} = \varvec{b}_u^r, \quad \varvec{A}_p^r\varvec{b} = \varvec{b}_p^r, \quad \varvec{A}_e^r\varvec{c} = \varvec{b}_e^r. \end{aligned}$$

Where $\varvec{A}_u^r = \varvec{\Phi }^\intercal \textbf{A}_u\varvec{\Phi } \in \mathbb {R}^{N_u\times N_u}$, $\varvec{A}_p^r = \varvec{\Xi }^\intercal \textbf{A}_p\varvec{\Xi } \in \mathbb {R}^{N_p\times N_p}$, and $\varvec{A}_e^r = \varvec{\Theta }^\intercal \textbf{A}_e\varvec{\Theta } \in \mathbb {R}^{N_e\times N_e}$. The resulting reduced linear systems in eq. (28) can be solved using any method for dense matrices. For example, the Householder rank-revealing QR decomposition of a matrix with full pivoting is used, and it is available in the Eigen library [42].

As the main idea here is to rely on a method capable of being as coherent as possible concerning the high-fidelity problem (Algorithm 1), in the following, the main steps for the reduced SIMPLE algorithm related to compressible turbulent flows are reported in Algorithm 1.

**Algorithm 1:**
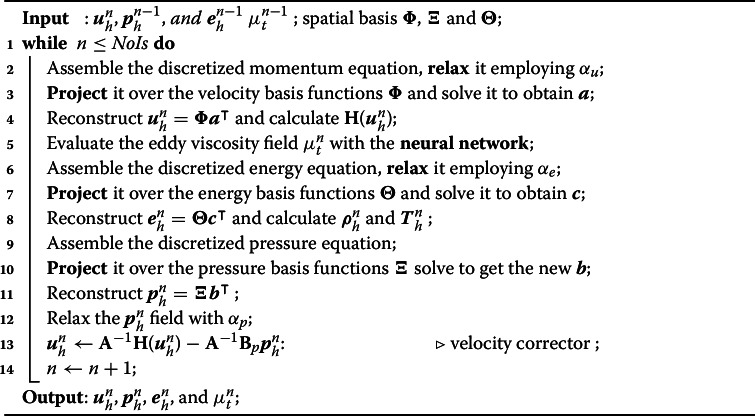
The reduced-SIMPLE algorithm

In Algorithm 1, $\textbf{H}(\varvec{u}_h^{n})$ is the extra-diagonal parts of the momentum matrix $\textbf{A}_u$ so that the following holds:

29 $$\begin{aligned} \textbf{A}_u \varvec{u}_h^{n} = \textbf{D}\varvec{u}_h^{n} - \textbf{H}(\varvec{u}_h^{n}), \end{aligned}$$

with $\textbf{D}$ being the diagonal part of $\textbf{A}_u$, and $\varvec{u}_h^{n}$ the velocities at iteration *n*. In addition, the *relaxation* of a quantity *Q* is given by:

30 $$\begin{aligned} Q^n = Q^{n-1} + \alpha (Q^{n*} - Q^{n-1}). \end{aligned}$$

Where $\alpha$ is the factor that defines the relaxation such that:

- $\alpha < 1$ means under-relaxation. This will slow down the convergence rate but increase the stability.
- $\alpha = 0$ means no relaxation at all. The predicted value of *Q* is simply used.
- $\alpha > 1$ means over-relaxation. It can sometimes be used to accelerate the convergence rate but will decrease stability.

$Q^n$ refers to the new used value, $Q^{n-1}$ the previous value, and $Q^{n*}$ the new predicted value. For more details, the interested reader can refer to OpenFOAM^®^ [43].

### Turbulence treatment

**Fig. 2:**
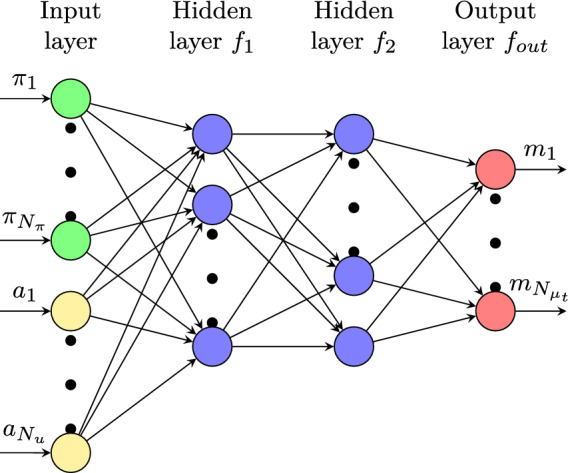
Schematic perspective of a fully connected neural network composed by an input layer, two hidden layers and an output layer, linking parameters $\pi _i$ and reduced velocity coefficients $a_i$ to predict the eddy viscosity coefficients $m_i$. $N_{\pi }$ being the number of parameters possibly existing in the problem

In this work some assumptions were taken in section “The compressible Navier–Stokes equations” leading to a simplified FANS system, Eq. 18. Turbulence effects in Eq. 18 are all due to the presence of the eddy viscosity field $\mu _t$. A technique has to be selected to model the eddy viscosity. Within this scope, many different approaches are possible [44–47].

To make our architecture as independent as possible on the turbulence model used during the offline phase to evaluate the $\mu _t$ field, this study combines a classical POD-Galerkin approach for what concerns the physical variables $\overline{p}, \tilde{\varvec{u}}$ and $\tilde{e}$ together with a data driven scheme for what concerns the eddy viscosity evaluation in the Boussinesq hypothesis [48].

Let us imagine to approximate the eddy viscosity field similarly to what has been done for all the other variables:

$$\begin{aligned} \mu _{t_r} = \sum _{i=1}^{N_{\mu _t}} m_i (\pi ) \varvec{\eta }_i (\varvec{x}), \end{aligned}$$

where $N_{\mu _t}$ is the number of basis functions selected to reconstruct/ predict the eddy viscosity field, $m_i$ are the coefficients depending only on the parameter $\pi$ while $\varvec{\eta }_i$ are the $\mu _t$ basis functions depend on the position $\varvec{x}$. During the offline phase, together with all the other saved solutions, also the eddy viscosity fields are exported and stored. Those snapshots are then collected into the $\varvec{S}_{\mu _t}$ matrix and used, as explained in , to obtain the requested basis functions. For what concerns the parameter coefficients $m_i$, they are evaluated through a Neural Network (NN) scheme linking the parameters of the problem $\pi _i$ and the reduced velocity coefficients $a_i$. In fact, it is well known that, no matter what turbulence model is employed, the eddy viscosity $\mu _t$ depends on the velocity field but, especially for geometrically parametrized problems, it also depends on the parameter itself. The reduced problem is thus completely independent of the choice of the turbulence model, and *step 2* into algorithm 1 can be performed efficiently. This would not have been the case if turbulence equations were projected. In case there was the necessity of changing the adopted turbulence model, all the architecture had to be modified.

In this work, we selected a fully connected Neural Network composed by an input layer, two hidden layers and an output layer. The input vector $\varvec{z}$ and output vector $\varvec{m}$ are defined as mentioned before:

$$\begin{aligned} \varvec{z}^\intercal = \begin{bmatrix} \pi _1, \cdots , \pi _{N_{\pi }}, a_1, \cdots , a_{N_u} \end{bmatrix}, \hspace{0.05cm} \varvec{m}^\intercal = \begin{bmatrix} m_1, \cdots , m_{N_{\mu _t}}, \end{bmatrix}. \end{aligned}$$

It is clear that the Neural Network has to be trained in some way. To this scope, the snapshots contained into $\varvec{S}_{\mu _t}$ are projected over their own basis functions $\varvec{\eta }_i$ to obtain the set of real coefficients $\{\varvec{m}_i\}_{i=1}^{N_t}$. They can be compared with the NN estimated coefficients $\{\tilde{\varvec{m}}_i\}_{i=1}^{N_t}$ into a loss function to target the training procedure. The loss function $\ell$ we adopted is a widely used quadratic one:

$$\begin{aligned} \ell = ||\varvec{m} - \tilde{\varvec{m}}||_{L^2}. \end{aligned}$$

The quantity $\mathcal {L}$ to be minimized during the training of the network is the sum of the loss function evaluated for all the different snapshots:

$$\begin{aligned} \mathcal {L} = \sum _{i=1}^{N_t} ||\varvec{m}_i - \tilde{\varvec{m}}_i||_{L^2}. \end{aligned}$$

The coefficients estimated by the network can be written as:

$$\begin{aligned} \tilde{\varvec{m}} = \varvec{\textit{f}}_{out} \left( \varvec{W}_{out} \, \varvec{\textit{f}}_2\left( \varvec{W_2} \varvec{\textit{f}}_1 \left( \varvec{W_1} \varvec{z} + \varvec{b}_1\right) + \varvec{b}_2\right) + \varvec{b}_{out} \right) , \end{aligned}$$

where $\varvec{\textit{f}}_1$, $\varvec{\textit{f}}_2$ and $\varvec{\textit{f}}_{out}$ are the activation functions, $\varvec{W}_1$, $\varvec{W}_2$ and $\varvec{W_{out}}$ are the weights while $\varvec{b}_1$, $\varvec{b}_2$ and $\varvec{b}_{out}$ are the biases, related to the first and the second hidden layers and to the output layer respectively. For the hidden layers, the best performing activation function appears to be the hyperbolic tangent, while the output layer has been simply implemented as a linear combination of the received data. The previous formula can then be simplified as follows:

$$\begin{aligned} \tilde{\varvec{m}} = \varvec{W}_{out} \, \tanh \left( \varvec{W}_2 \, \tanh \left( \varvec{W}_1 \varvec{z} + \varvec{b}_1\right) + \varvec{b}_2\right) + \varvec{b}_{out}, \end{aligned}$$

where $$\tanh (\varvec{y})^\intercal = \begin{bmatrix} \tanh (y_1), \cdots , \tanh (y_{dim_y}) \end{bmatrix}$$, being $\varvec{y} = [y_1, \ldots , y_{dim_y}]$ a generic vector quantity.

## Numerical results

In this section, we first discuss the mesh characteristics with boundary conditions. After that, we discuss the two types of parametrization here considered. Firstly a physical parametrization on the viscosity, and secondly a geometrical parametrization on the airfoil’s shape.

### Definition of the test case

For a wall boundary condition, $\varvec{u}$ is assigned a noSlip condition, *p* and *T* are assigned a zero gradient. $\omega$ is assigned a omegaWallFunction, eddy viscosity is also assigned a nutkWallFunction, and *k* is assigned a kqRWallFunction. The values at the inlet of the turbulence quantities ($\nu _t$, *k*, and $\omega$) are set in a way to have already at the inlet a very small turbulent viscosity in order to resample the low turbulence intensity of the domain [49]. The boundary conditions applied are summarized in Table 1.

**Table 1:** A summary of the boundary conditions OpenFOAM^®^ [43]

	**Inlet**	**Outlet**	**Airfoil**
$\varvec{u}$	freestreamVelocity	freestreamVelocity	no Slip
*p*	freestreamPressure	freestreamPressure	zero Gradient
*T*	inletOutlet	inletOutlet	zero Gradient
*k*	inletOutlet	inletOutlet	kqRWallFunction
$\omega$	inletOutlet	inletOutlet	omegaWallFunction
$\nu _t$	calculated	calculated	nutkWallFunction

For the pressure at the inlet, the freestreamPressure boundary conditions are used. It is an outlet-inlet condition that uses the velocity orientation to continuously blend between zero gradient for normal inlet and fixed value for normal outlet flow. The freestreamVelocity velocity boundary condition switches between fixed Value and zero Gradient depending if the mass flux points inside (fixed value) or outside (zero Gradient) the domain.

**Fig. 3:**
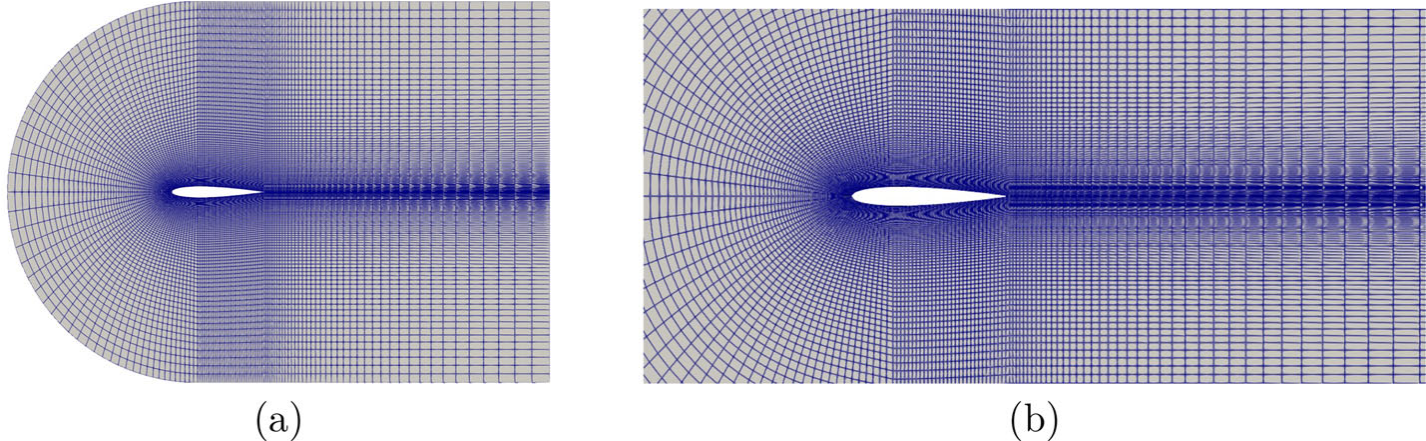
**a** Picture showing the computational domain and the mesh around the foil with 16,000 cells (control volumes) and 32 480 node points with a chord length 1.0 m, **b** a near view mesh around the airfoil

### Physical parametrization test case

The first test case we present in this work is a physical parametrization of an external flow. A NACA0012 airfoil is immersed into a fluid with variable viscosity $\mu$. The unperturbed velocity is fixed and is equal to $\tilde{\varvec{u}}_{inlet}=[ 250, 0, 0]^T$ m/s while the chord of the airfoil is equal to one. As already said, the viscosity can vary so that $\mu \in [10^{-5}, 10^{-2}]$. The speed of sound at the inlet can easily be evaluated by taking into consideration the thermophysical properties of the gas we are working with. We consider perfect gases. Thus, the specific heat transfer at constant pressure is sufficient to evaluate $\gamma = \frac{C_p}{C_v} = \frac{C_p}{C_p-R}$ where $C_p=1005{\hbox {Jkg}}\,{\hbox {K}}^{-1}$ while $R=8,314 {\hbox {mol}}\,{\hbox {K}}^{-1}$ is the constant for perfect gases. The airfoil is supposed to move into air so that $M=28,9 {\hbox {g}}\,{\hbox {mol}}^-{1}$ where *M* stands for the molar weight. Temperature is fixed at $T=298\,{\hbox {K}}$. Collecting all these data together, we end up with $C = \sqrt{\frac{\gamma R T}{M}} = 341.17 {\hbox {m}}\,{\hbox {s}}^{-1}.$ This means that at the inlet the Mach number can be calculated as

$$\begin{aligned} \text {Mach}=\frac{\tilde{\varvec{u}}_{inlet}}{C} \simeq 0.73. \end{aligned}$$

For this test case, consequently, a compressible treatment for the flow is needed since we are approaching the *Transonic regime* and compressible effects are pretty significant. At the inlet, pressure is fixed to $10^5$ Pa. Then the Reynolds number can be evaluated as

$$\begin{aligned} \text {Re}=\frac{\rho L \tilde{\varvec{u}}_{inlet}}{\mu }=\frac{p L \tilde{\varvec{u}}_{inlet} M}{\mu R T}. \end{aligned}$$

The resulting Reynolds number is then $\text {Re}\in 2.92 \times \left[ 10^4, 10^7 \right]$, which clearly requires treatment for turbulence since the system is operating in a fully turbulent regime.

For the offline phase, 50 random values have been selected: $\pi _i \in [10^{-5}, 10^{-2}] \ \text {for} \ i=1,\ldots ,50$ where $[\pi _1, \ldots , \pi _{50}]=\mathbb {P}$. Full-order eddy viscosity is calculated by the resolution of a $k-\omega$ turbulence model [38].

**Fig. 4:**
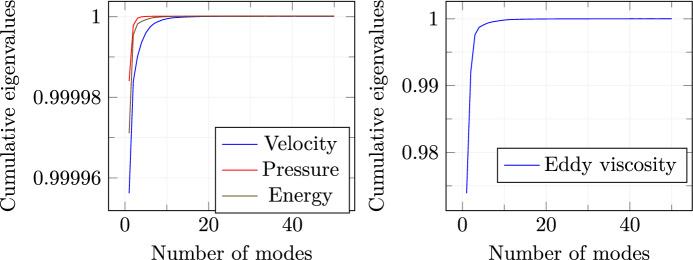
Cumulative eigenvalues trends

Figure 4 shows the trends of the *cumulative* eigenvalues for velocity, pressure, energy, and eddy viscosity. As we may notice, by just considering a few modes for every variable, the amount of discarded information is pretty low. For this reason, just the first 20 modal basis functions have been selected for velocity, pressure, and energy while 30 modal basis functions are used to predict the eddy viscosity field. This is due to the fact that by analyzing Fig. 4, it can be noticed that a higher number of basis functions are needed in order to approach the unity in the cumulative eigenvalues plot. With these numbers of modes, the cumulative variance reach approximately 99.999%.

As explained in subsection “Turbulence treatment”, the neural network for the eddy viscosity coefficients employed here is composed by: an input layer, whose dimension is equal to the dimension of the reduced velocity, i.e. 20, plus one for the parameter, two hidden layers of dimension 256 and 64 respectively, an output layer of dimension 30 for the reduced eddy viscosity coefficients. The net is a fully connected one. Moreover, the neurons of the hidden layers are characterized by the employment of $\tanh$ activation functions. For the training procedure, the Adam optimizer has been selected with a learning rate set to $10^{-4}$. The training procedure is carried out in $2 \times 10^4$ epochs. The training set is composed by both the intermediate and final solutions obtained during the offline phase, randomly selected. To control the training procedure, a test set has also been selected. 20 totally random new parameter values have been chosen, and their related full solutions have been calculated, saving both final and intermediate steps, coherently with the offline snapshots used for training. The conditions on which this model performs well depends with high-quality, representative, and sufficient amount of data. On the contrary, with poor data quality and small datasets, the model could have some limitations.

**Fig. 5:**
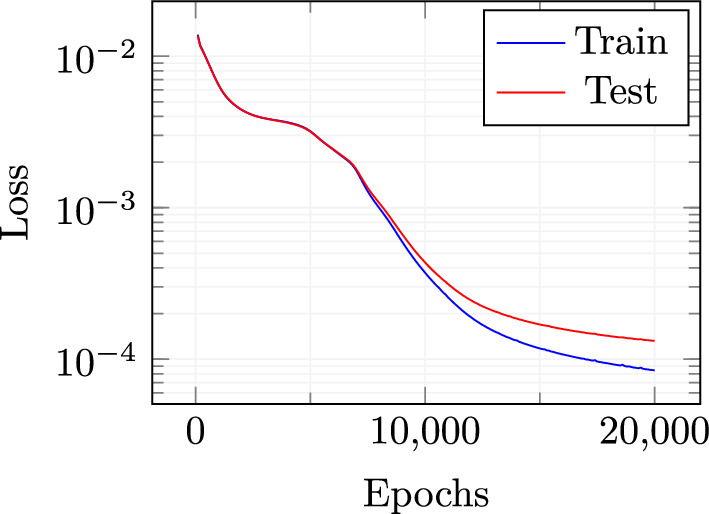
Loss function decay for both train and test sets

A mean squared error loss function is used to evaluate the prediction capability of the network for both training and testing sets. The decay behaviour of both losses is depicted in Fig. 5. The training stage was stopped after $2 \times 10^4$ epochs to avoid over-fitting as the distance between test and train losses was starting to increase significantly.

**Fig. 6:**
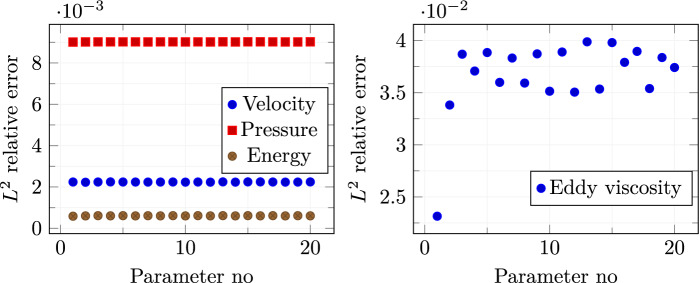
$L^2$ norm relative errors

Figure 6, left, shows the $L^2$ norm relative errors for all the different parameters in the online set concerning velocity, pressure, and internal energy. Figure 6, right, shows the $L^2$ norm relative error for the eddy viscosity between full-order and reduced-order for the whole online parameter set. As we may notice, even if the order of magnitude of the $\nu _t$ error is equal to $10^{-2}$, it is sufficient to ensure a lower error for the quantities of interest, i.e. velocity, pressure, and energy. By this observation we are allowed to employ such a small neural network which is not compromising the computational cost, still ensuring good performances.

**Fig. 7:**
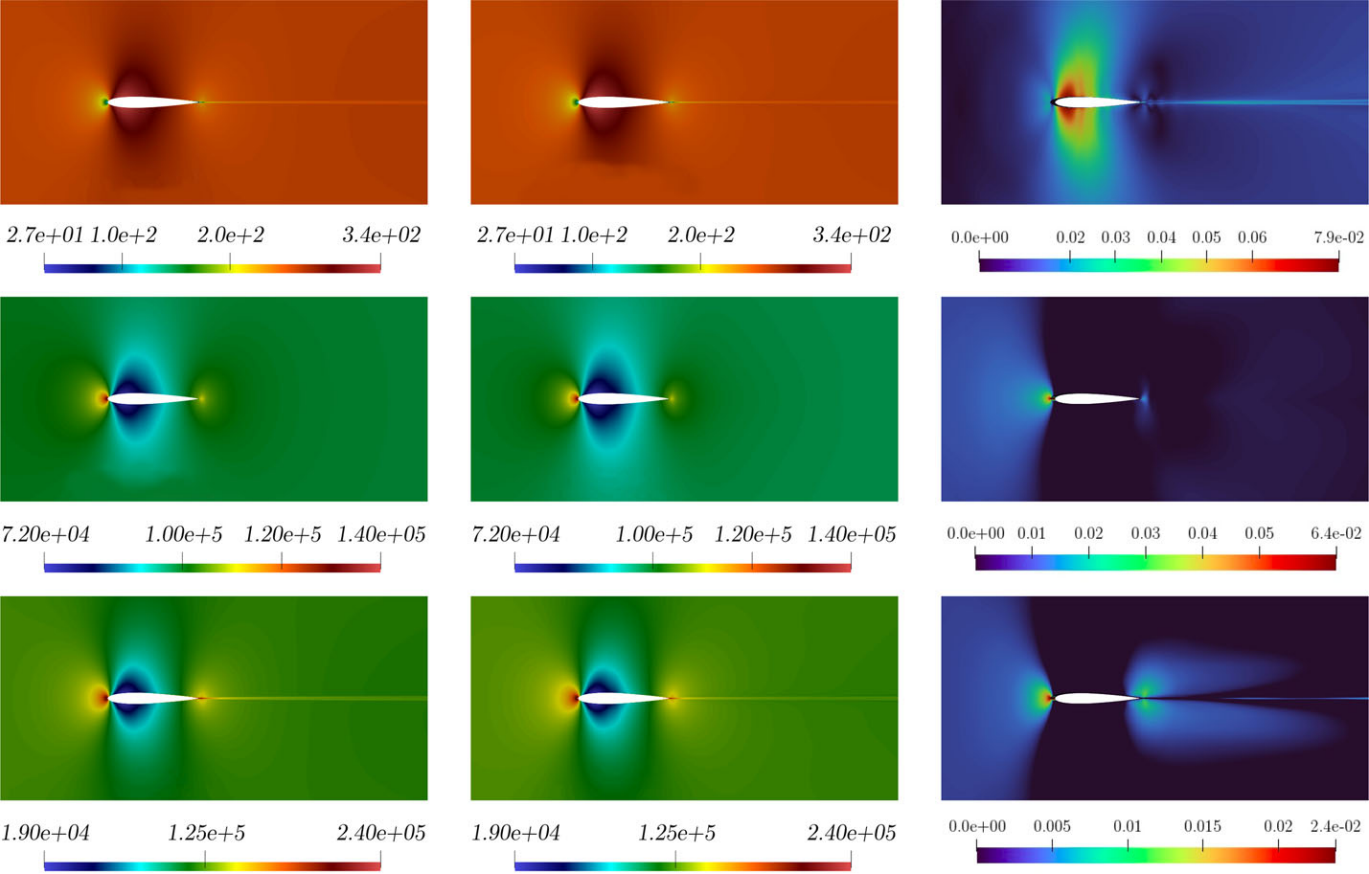
Comparison between full-order (first column), reduced-order solutions (second column), and relative error associated (third column). *Velocity* (first row), *pressure* (second row), and *energy* (third row) magnitude. These fields refer to the resolution of the problem for $\pi = \mu = 0.21 \times 10^{-3}$ which has been selected as a random value in the online parameter set

**Fig. 8:**
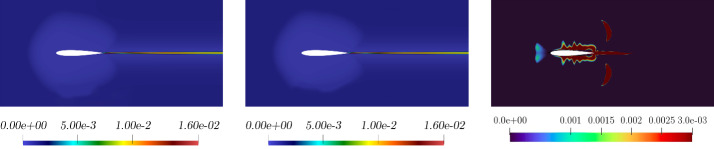
Comparison between full-order (left picture), reduced-order (middle picture) for the *eddy viscosity* solutions, and relative error associated (third picture). These fields refer to the resolution of the problem for $\pi = \mu = 0.21 \times 10^{-3}$ which has been selected as a random value in the online parameter set

In Fig. 7 and Fig. 8 a comparison between full-order and reduced-order solutions is depicted, for a random value of the parameter, included in the online set. By analysing the depicted fields, full-order and reduced-order solutions appear to be very similar, and the most important areas in the domain, i.e. the zone surrounding the airfoil together with the wake created by the body, are well predicted. Additionally, the related error confirms the above observation.

### Geometrical parametrization test case

**Fig. 9:**
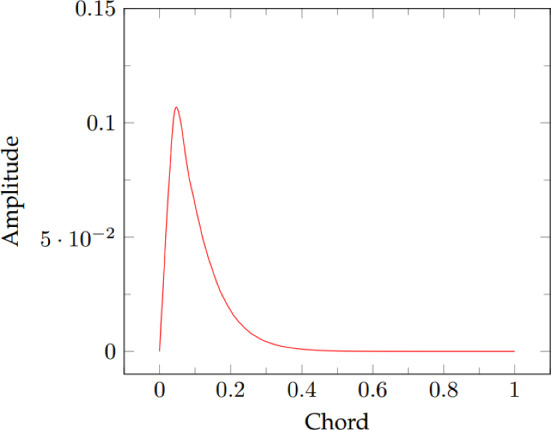
Shape of the employed bump function

This section presents the second test case, focused on a geometrically parameterized problem. The shape of the airfoil used into subsection “Multiscale modeling” is modified by the use of a bump function. In particular, the foil is divided in a top and a bottom part by the chord. The bump function depicted in Fig. 9 is added to the top and subtracted to the bottom surface, premultiplied by two different amplitude scalar factors: every solution is parameterised uniquely by two different scalar values. We used the same thermophysical properties used for subsection “Multiscale modeling” but the dynamic viscosity is fixed and equal to $1.74 \times 10^{-5} Pa \, s$. Moreover, the inlet velocity has been slightly decreased since the random modification of the geometry may lead to high curvature areas where the flow could eventually become supersonic: $$\tilde{\varvec{u}}_{inlet}=\begin{bmatrix} 170, 0, 0 \end{bmatrix}^T {\hbox {m}}\,{\hbox {s}}^{-1}$$. This means that the Mach number at the inlet is now around 0.5. For the offline phase, 50 random values have been selected: $\pi _{top_i}, \pi _{bottom_i} \in [0,0.1]$ for $i= 1,...,50$ where $\mathbb {P} = \{(\pi _{top_i}, \pi _{bottom_i}) \}_{i=1}^{50}$. The full-order eddy viscosity is calculated by the resolution of a $k-\omega$ turbulence model [38].

The general POD approach described in subsection “Proper Orthogonal Decomposition procedure” is not directly applicable to a geometrical parametrization problem, since the $L^2$-norm used for the inner products is not well-defined in case of multiple different domains. The mesh in our case is moved thanks to a Radial Basis Functions (RBF) algorithm where the points on the moving boundaries are displaced by the application of the desired law and their displacements are used as boundary conditions for an interpolation procedure, performed in order to move all the remaining points of the grid. The interested reader may find a deeper explanation of this technique in [50] or some applications in [51] and [52]. By exploiting the aforementioned method, the mesh is modified for each offline solution. To take into account the fact that all the snapshots are defined over a different mesh, the grid is taken back to its undeformed state before starting the POD procedure: the mass matrix we consider evaluating the norms is then the reference unperturbed one.

**Fig. 10:**
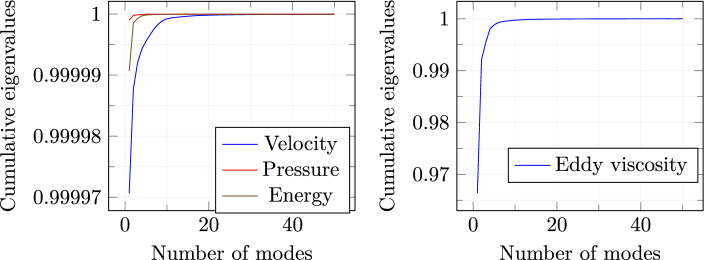
Cumulative eigenvalues trends

To test the online performances, 20 new scalar amplitude couples have been randomly selected. 30 modal basis functions have been picked for the prediction of velocity, pressure and internal energy fields, while 15 modal basis functions have been employed for $\nu _t$. This choice is supported by what is shown in Fig. 10 i.e. the increasing trend of the cumulative eigenvalues is pretty fast and this fact allows the discarding of the modes higher than the fixed quantity. For every new parameter couple, the mesh motion has to be performed, but the procedure is very efficient since the coefficients for the RBF have to be evaluated and stored just once [52].

**Fig. 11:**
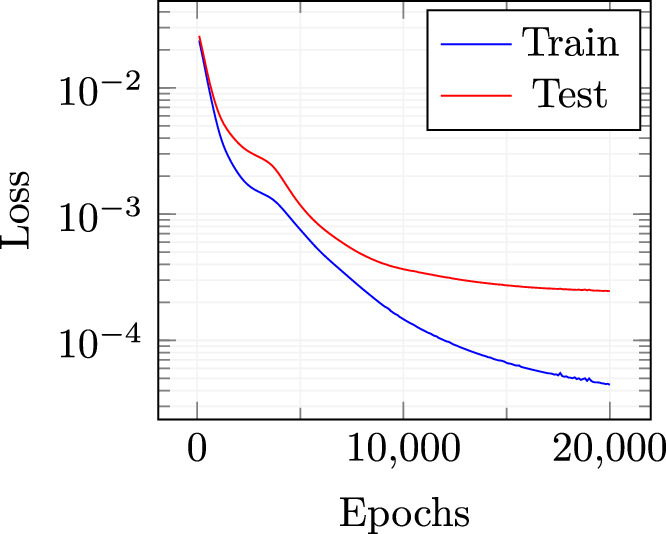
Loss function decay for both train and test sets

The same neural network used for Fig. 14 is employed here for what concerns the eddy viscosity. Again, looking at Fig. 11, it can be noticed that the learning of the net seems to stabilize after $2\times 10^4$ epochs which is the threshold we fixed for the training procedure.

**Fig. 12:**
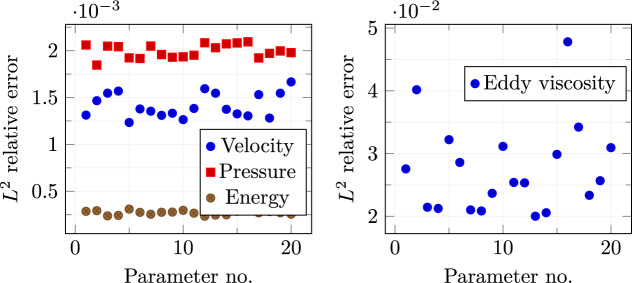
$L^2$ norm relative errors

The resulting $L^2$ norm errors for all the parameter couples in the online set are shown in Fig. 12. Once again, a discrepancy of about one order of magnitude can be noticed between the relative errors for the quantities of interest and the one calculated for the eddy viscosity. This is because we are using a very simple and small network, but it reveals to be reliable enough to make the online algorithm work fine.

**Fig. 13:**
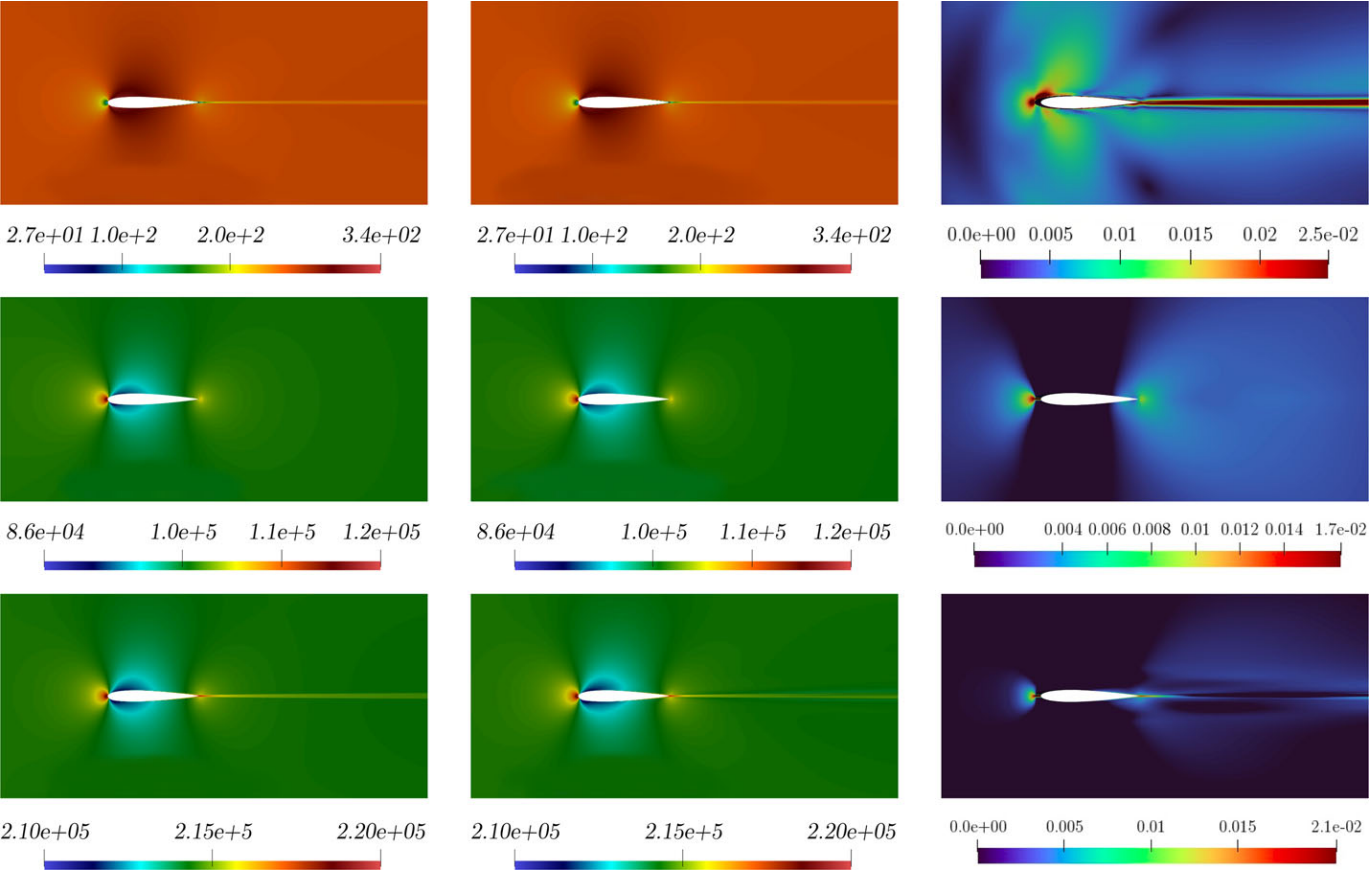
Comparison between the full-order (first column) and reduced-order (second column) solutions with the relative error associated (third column): *velocity* (first row), *pressure* (second row), and *energy* (third row) magnitude. These fields refer to the resolution of the problem for $(\pi _{top}, \pi _{bottom}) \simeq ( 0.004,0.086)$ which has been selected as a random value in the online parameter set

**Fig. 14:**
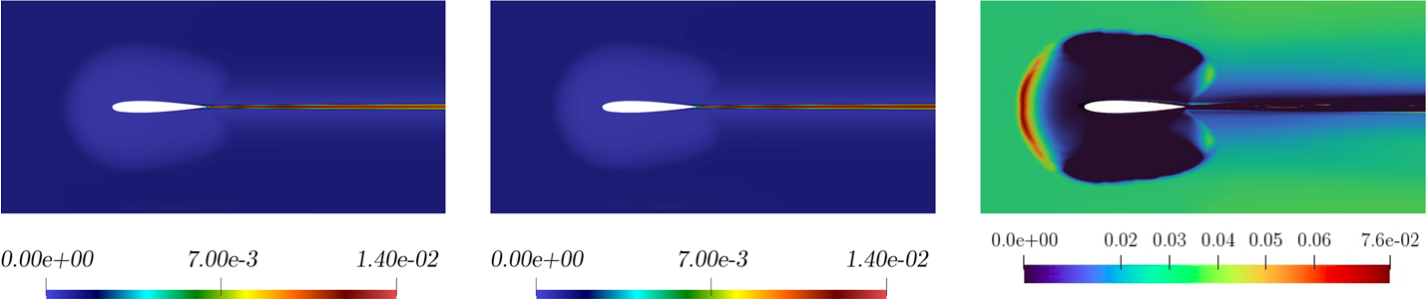
Comparison between the full-order (left picture) and reduced-order (middle picture) with the relative error associated (right picture) for the *eddy viscosity* solutions. These fields refer to the resolution of the problem for $(\pi _{top}, \pi _{bottom}) \simeq ( 0.004,0.086)$ which has been selected as a random value in the online parameter set

**Fig. 15:**
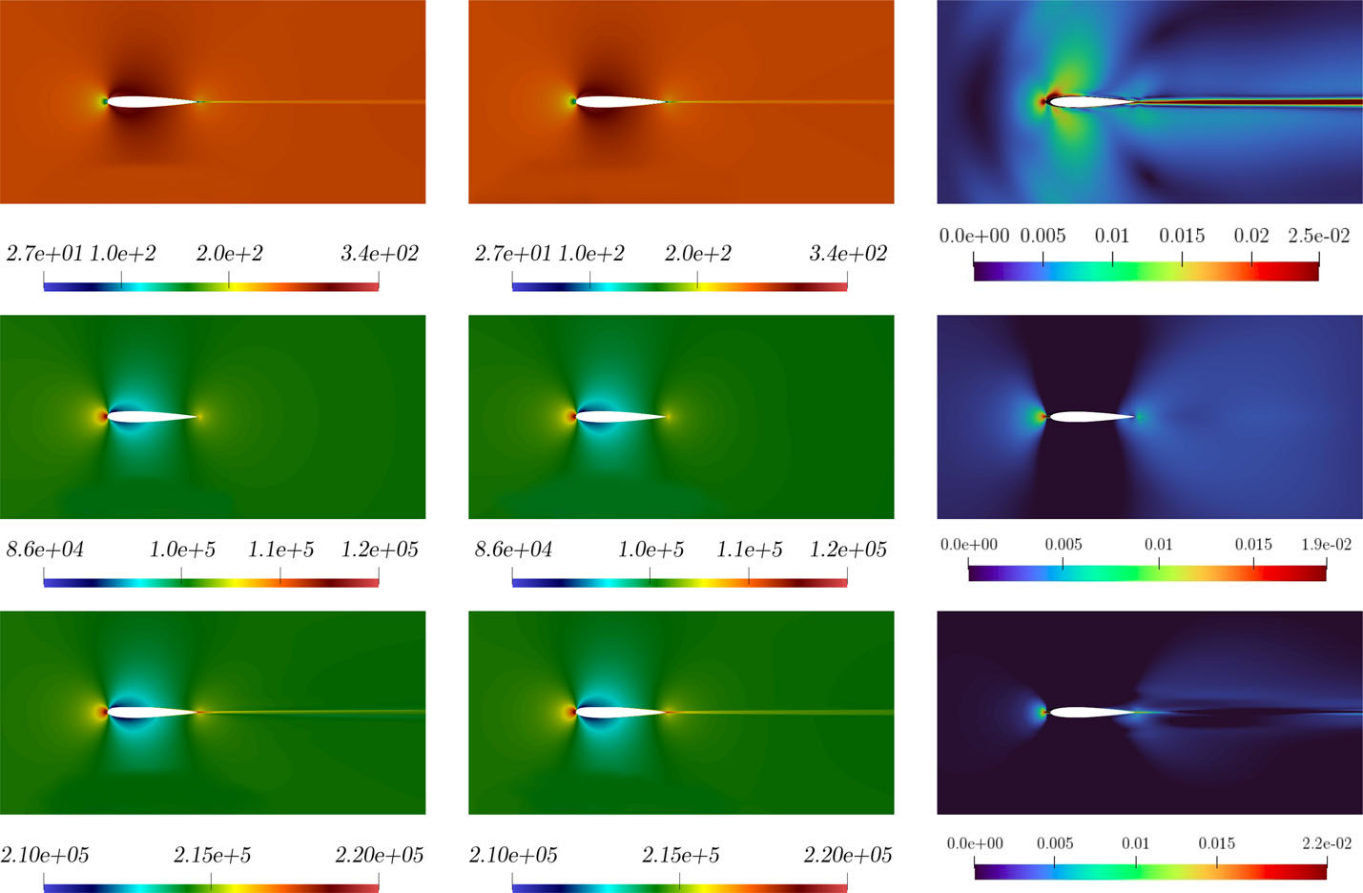
Comparison between the full-order (first column) and reduced-order (second column) solutions, with the relative error associated (third column): *velocity* (first row), *pressure* (second row), and *energy* (third row) magnitude. These fields refer to the resolution of the problem for $\pi _{top}\simeq 0.095$ and $\pi _{bottom}\simeq 0.003$ which has been selected as a random value in the online parameter set

**Fig. 16:**
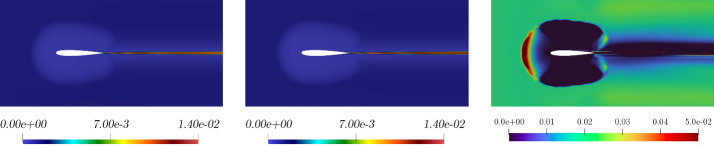
Comparison between the full-order (left picture) and reduced-order (middle picture) with the relative error associated (right picture) for the *eddy viscosity* solutions. These fields refer to the resolution of the problem for $(\pi _{top}, \pi _{bottom}) \simeq (0.095,0.003)$ which has been selected as a random value in the online parameter set

In Fig. 13, Fig. 14, Fig. 15 and Fig. 16 a comparison between offline and online solutions and the error field associated are presented for two different parameter couples selected from the online set. Even if the two solutions are obtained for airfoil geometries that are perturbed in opposite directions, in both cases the method exhibits good reliability properties even though the intermediate solutions introduced in the snapshots matrix could be highly inaccurate and trigger instabilities in the ROMs. Additionally, Fig. 2 reports an error analysis versus different number of modes. This allows for evaluating the effect of the number of modes variation on the error between the full and reduced order solutions of all the field of interests.

**Table 2:** Error comparison versus the # modes for ($\pi _{top}, \pi _{bottom} ) \simeq (0.055, 0.0051).$

**#** * of modes*	$\mathbf{{L}}^\textbf{2}$ **relative error**			
	$\varvec{u}$	$\varvec{p}$	$\varvec{e}$	$\varvec{\nu }_{\varvec{t}}$
$N_u=N_{{\nu }_t}=10$ *and* $N_p=N_{e} = 2$	0.018	0.00311	0.0173	0.652
$N_u = N_{{\nu }_t} = 10$ *and* $N_p = N_{e} = 5$	0.0163	0.0044	0.0034	0.612
$N_u=N_{p} = 20$ *and* $N_e = N_{{\nu }_t}=30$	0.0118	0.0033	0.0019	0.284
$N_u = N_p = N_{e} = 20$ *and* $N_{{\nu }_t}=30$	0.0114	0.00320	0.00206	0.2784

Regarding the computational cost of the reduced-order model, it should be noted that the solution of the reduced system is not proportional to the reduction of the unknowns obtained at the reduced-order level. This is due to the fact that in the presence of a deforming domain such as the one considered in the second case, the entries of the matrices of the ROM system must be computed at each iteration through integrals on the updated full-order grid. Clearly, this is at the moment represents a major bottleneck towards a ROM which grants significant computational cost reduction with respect to its FOM counterpart, and work is being carried out towards lowering the computational cost associated with the reduced model assembling using hyper-reduction techniques [53, 54]. Nonetheless, the main goal of the present work is that of assessing the accuracy of the ROM approach taken. In particular, it is important establishing whether the interaction between the physics based reduction of the fluid dynamic balance equations, and the data driven reduction of the turbulence and shape deformation results in an accurate solver.

## Conclusions and future perspectives

This study focused on compressible flows by proposing a new mixed technique, capable of merging the reliability of Galerkin-projection methods together with the versatility of data-driven strategies in turbulence and compressible flows. The good results obtained for both a physical and geometrical parameterized benchmarks make this approach quite promising. From one hand, the possibility to freely select the turbulence model avoiding the necessity of changing the whole architecture is attractive, while on the other hand, the guarantee of a strong connection with physical aspects given by the projection of conservation laws is reassuring.

The segregated compressible algorithm, proposed in subsection “Multiscale modeling”, also introduces a way to provide accurate reduced solutions without any kind of stabilization: the employment of a decoupled approach for the compressible turbulent Navier–Stokes equations relies on the chipping of the saddle point formulation. For this reason, no stabilization for pressure is required: as shown in both subsection “Multiscale modeling” and subsection “Geometrical parametrization test case”, pressure field solutions do not exhibit significant instability or inaccuracy issues. This aspect helps the procedure on being more consistent without pollution of the resulting solution due to stabilization. A natural extension of this work will be a deep analysis with others existing approaches both in the methodology and application. Another extension will be the application of neural networks to approximate the functional evaluations required by the online phase to overtake the necessity of reconstructing the full fields at each iteration. This aspect would increase the performances but it has to be carefully calibrated to avoid possible drifting of the algorithm resulting on the loss of the convergence.

A final aspect that can be improved is the neural network itself: a weighted strategy where eigenvalues play a relevant role in the loss function would, in principle, enhance the training stage since the first modal basis functions, represented by the highest eigenvalues, are the most significant ones on the reconstruction procedure.

## Data Availability

The data sets generated during and/or analyzed during the current study are available from the corresponding authors on reasonable request.
